# Agreement of glomerular filtration rate estimation equations for chemotherapy dosing in cancer patients at a tertiary referral hospital in Sub-Saharan Africa

**DOI:** 10.1371/journal.pone.0325883

**Published:** 2025-06-12

**Authors:** Wubshet Jote Tolossa, Tigist Workneh Leulseged, Abdu Adem, Feyissa Challa, Tirumebet Mezgebu, Ruth S. Aytehgeza, Nebiat Adane Mera, Kalsidagn Girma Asfaw, Momina M. Ahmed, Kebede H. Begna

**Affiliations:** 1 Department of Internal Medicine, Menelik II Comprehensive Specialized Hospital, Addis Ababa, Ethiopia; 2 Department of Internal Medicine, St. Paul’s Hospital Millennium Medical College, Addis Ababa, Ethiopia; 3 Medical Research Lounge (MRL), Addis Ababa, Ethiopia; 4 National Reference Laboratory for Clinical Chemistry, Ethiopian Public Health Institute (EPHI), Addis Ababa, Ethiopia; 5 Department of Internal Medicine, Yekatit 12 Hospital Medical College, Addis Ababa, Ethiopia; 6 Department of Emergency Medicine and Critical Care, Addis Ababa University, College of Health Sciences, Addis Ababa, Ethiopia; 7 King Faisal Hospital, Kigali, Rwanda; 8 Department of Internal medicine, Division of Hematology, Mayo Clinic, Minnesota, United States of America; Memorial Sloan Kettering Cancer Center, UNITED STATES OF AMERICA

## Abstract

**Introduction:**

Narrow therapeutic indices of chemotherapeutic agents necessitate precise dosing to ensure efficacy and minimize nephrotoxicity. Due to the complexity of directly measuring Glomerular filtration rate (GFR), renal dosing is usually based on GFR estimation equations. The Cockcroft-Gault formula remains the most widely used equation in cancer patients, despite the availability of more precise kidney function estimation equations. Therefore, the aim of the study was to assess the agreement between Cr and cystatin-C (CysC) based GFR estimation equations and GFR estimated by Cockcroft-Gault for appropriate chemotherapy dosing in cancer patients undergoing assessment for first-Line chemotherapy at an oncology unit of St. Paul’s Hospital Millennium Medical College in Ethiopia.

**Methods:**

A cross-sectional study was conducted among 136 adult cancer patients scheduled to initiate chemotherapy at the hospital between November 1, 2021, and April 30, 2022. GFR was calculated using 12 different GFR estimation equations to be compared with Cockcroft-Gault; MDRD, MDRD adjusted for ethnic factor (MDRDef), the 2009 CKD-EPI calculated based on serum creatinine (CKD-EPI 2009 Cr), the 2009 CKD-EPI Cr adjusted for ethnic factor (CKD-EPI 2009 Cref), the 2012 CKD-EPI calculated based on serum Cystatin C (CKD-EPI 2012 CysC), the 2021 CKD-EPI calculated based on serum creatinine and Cystatin C (CKD-EPI 2021 Cr-CysC), the 2021 CKD-EPI (CKD-EPI 2021), FAS calculated based on serum creatinine (FAS Cr)¸ FAS Cr adjusted for African coefficient (FAS Craf), FAS calculated based on serum Cystatin C (FAS CysC), FAS calculated based on serum creatinine and Cystatin C (FAS Cr-CysC), and FAS Cr-CysC adjusted for African coefficient (FAS Cr-CysCaf). To assess the level of agreement, bias (mean error/ME), precision, and accuracy (root-mean squared error/ RMSE) were analyzed for each equation, where for all measurements a value closer to 0 indicates minimal bias, high precision, and high accuracy demonstrating good agreement with Cockcroft-Gault. To confirm the significance of the recorded levels of agreement, a one-sample t-test, a Bland-Altman plot, and a linear regression analysis were performed step by step for variables which proved to have statistical agreement, where a p-value > 0.05 and the presence of heteroscedasticity indicates a non-significant difference and hence the presence of good agreement.

**Results:**

The GFR estimation equations revealed variation, with some methods underestimating and others overestimating GFR. However, only four equations showed potential agreement with Cockcroft-Gault based on a one-sample t-test: MDRD, CKD-EPI 2009 Cr, CKD-EPI 2021, FAS Cr, and FAS Cr-CysCaf. Among these, CKD-EPI 2009 Cr exhibited the least bias (ME = 0.72 ml/min, 95% CI: −67.66, 69.10 ml/min), while FAS Cr-CysCaf demonstrated the highest precision (SD = 33.92) and accuracy (RMSE = 34.53). However, further analysis using Bland-Altman plots and linear regression to confirm agreement revealed no agreement between any of the formulas and Cockcroft-Gault.

**Conclusion:**

The study revealed that the most recent and accurate GFR estimation equations that are recommended to be used in cancer patients did not show agreement with Cockcroft-Gault. This suggests that current GFR estimation practices in cancer patients might be inaccurate, potentially leading to improper chemotherapy dosing and poorer patient outcomes.

## Introduction

Glomerular filtration rate (GFR) is routinely used to quantify kidney function and diagnose and follow kidney dysfunction. While directly measured GFR using exogenous filtration markers like Inulin, Iothalmate, Iohexol, and radiocontrast agents like chromium-51 labeled ethylenediamine tetra acetic acid (51Cr-EDTA) provides the most accurate assessment, these methods are complex, time-consuming, costly, and impractical for routine clinical use [1]. To address this, various formulas have been developed to estimate GFR using endogenous filtration markers like serum creatinine, urea, and cystatin C [1–4].

GFR estimation is crucial for clinicians to guide selection of appropriate drugs, determine appropriate dosing, assess eligibility for interventional drugs, and predict treatment outcomes of patients [2]. This is particularly important in cancer patients because many anticancer drugs are excreted through the kidneys and have narrow therapeutic windows, and a significant number of cancer patients experience impaired GFR due to factors like old age, gastrointestinal loss, dehydration, and comorbid illnesses [2,5–7]. Additionally, cancer patients often present with underlying kidney function impairment, with an estimated GFR (eGFR) of less than 90 ml/min/1.73m² in over half and less than 60 ml/min/1.73m² in up to one-fifth of patients with solid tumors [2,5,8]. Moreover, up to 15% of patients with eGFR less than 60 ml/min/1.73m² may have a normal creatinine value [5,6]. Therefore, using serum creatinine alone to assess kidney function may overestimate kidney function, predisposing patients to nephrotoxicity. Hence, the International Society of Geriatric Oncology and the National Comprehensive Cancer Network recommend assessing kidney function to adjust drug dosage even when serum creatinine is within the normal range [6,7,9,10].

The first GFR estimation equation, the Cockcroft-Gault formula, was developed in 1976 and remains widely used despite the emergence of more accurate equations in the past two decades [4,6,7,11–13]. These newer equations include the 1999 Modification of Diet in Renal Diseases (MDRD) [14], the 2009 Chronic Kidney Disease Epidemiology Collaboration (CKD-EPI 2009), the 2021 Chronic Kidney Disease Epidemiology Collaboration (CKD-EPI 2021) [15,16], and the 2016 Full Age Spectrum (FAS) [17]. The Kidney Disease Improving Global Outcome (KDIGO), American Society of Nephrology (ASN) and National Kidney Foundation (NKF) currently recommend the 2021-CKD EPI formula to calculate GFR and stage chronic kidney disease. Concerning drug dosing, the general recommendation is to adopt newer, more accurate equations as they evolve. [18,19] For cancer chemotherapy, the recommendation is using the most accurate available tool for assessing kidney function in drug dosing. This is highly dependent on local experience and availability [20]. However, the continued use of the Cockcroft-Gault formula in cancer patients presents challenges. Many anti-cancer drug cutoffs were determined before the development of these more accurate equations and the standardization of creatinine assays across different populations and environments. This has complicated the decision-making process regarding which equation to use. Consequently, there are no universal guidelines on the preferred method for estimating kidney function in cancer patients, including the need to adjust for race for estimating GFR in the Sub-Saharan African population [6–8,10,21].

Studies comparing different GFR estimation equations in cancer patients have yielded varying results, with different equations found to be more accurate in different studies [19–21]. Furthermore, one study found that adjusting for African-American ethnicity did not improve the performance of GFR estimation equations [22].

While there is no study specifically validating GFR estimation equations for Ethiopian cancer patients, some studies done in other patient groups have shown relevant results. A study conducted on HIV-positive and -negative patients showed no difference between the eGFR of the two groups. However, removing the ethnic coefficient reduced the bias and improved the accuracy of CKD-EPI 2009 and MDRD estimation equations [23]. Another study on patients with type 2 diabetes mellitus revealed that cystatin C-based GFR estimation equations detect renal impairment earlier than creatinine-based equations [24].

Population-based surveys in Ethiopia indicate an increase in the prevalence of major risk factors for chronic kidney disease and an 11% risk of developing cancer before the age of 75 [25,26]. This suggests that with a growing population at risk, having a proper way of estimating GFR is crucial. Yet, there is no study validating GFR estimation equations for this specific population group in this setting. Therefore, the aim of the study was to assess the agreement between creatinine- and cystatin-C-based GFR estimation equations and GFR estimated by Cockcroft-Gault for appropriate chemotherapy dosing in cancer patients undergoing assessment for initiation of first-line chemotherapy at an oncology unit of a tertiary referral hospital in Ethiopia.

## Materials and methods

### Study setting and design

An institution-based, analytic cross-sectional study was conducted at St. Paul’s Hospital Millennium Medical College (SPHMMC) in Addis Ababa, Ethiopia, from November 1, 2021, to April 30, 2022. SPHMMC is a rapidly expanding medical college with a large, tertiary-level healthcare hospital. The hospital has an inpatient capacity of over 700 beds and an average of 1200 emergency and outpatient visits daily. The hospital provides services in various specialty fields, including Oncology and Hematology, on an outpatient and inpatient basis.

### Population and sample size

The study included all new adult cancer patients (solid and hematologic malignancies) diagnosed and scheduled to initiate chemotherapy for the first time at the hospital between November 1, 2021, and April 30, 2022. During this study period, a total of 140 patients commenced chemotherapy at the hospital. Of these, only 136 met the inclusion criteria. Four patients were excluded from the study as they were already started on chemotherapy upon transfer to our hospital. Due to the small sample size of eligible patients, all 136 cases were included in the final analysis.

### Data collection procedures and quality assurance

Data was collected using a pre-tested, structured interviewer-administered electronic questionnaire which contained questions on socio-demographic characteristics, weight, height, oncologic diagnosis, and planned chemotherapy agents. Furthermore, serum creatinine was done for all 136 patients. However, due to reagent unavailability, Cystatin C measurements were done for only 81 patients that were randomly selected from the 136 cases. Accordingly, all GFR estimation equations calculated using Cystatin C were compared with the standard based on these 81 patients exclusively. To ensure that no significant differences existed in the underlying characteristics of the cases for which Cystatin C was measured and those for which it was not, a chi-square test (Fisher’s exact test where appropriate) was run. Accordingly, there were no statistically significant differences observed between the groups in terms of age, sex, Body Mass Index (BMI), and the prevalence of chronic medical illnesses, including hypertension, type 2 diabetes mellitus, human immunodeficiency virus (HIV), Anemia, chronic kidney disease (CKD), asthma, pulmonary tuberculosis, and deep venous thrombosis/pulmonary thromboembolism (DVT/PTE).

Cockcroft-Gault equation estimated GFR was used for drug dosing as per the hospital’s protocol and used as a reference to assess the performance of the other estimations. Based on the serum creatinine and cystatin C values, GFR was calculated using 12 different GFR estimation equations to be compared with Cockcroft-Gault; MDRD, MDRD adjusted for ethnic factor (MDRDef), the 2009 CKD-EPI calculated based on serum creatinine (CKD-EPI 2009 Cr), the 2009 CKD-EPI Cr adjusted for ethnic factor (CKD-EPI 2009 Cref), the 2012 CKD-EPI calculated based on serum Cystatin C (CKD-EPI 2012 CysC), the 2021 CKD-EPI calculated based on serum creatinine and Cystatin C (CKD-EPI 2021 Cr-CysC), the 2021 CKD-EPI (CKD-EPI 2021), FAS calculated based on serum creatinine (FAS Cr)¸ FAS Cr adjusted for African coefficient (FAS Craf), FAS calculated based on serum Cystatin C (FAS CysC), FAS calculated based on serum creatinine and Cystatin C (FAS Cr-CysC), and FAS Cr-CysC adjusted for African coefficient (FAS Cr-CysCaf).

Data was collected by four oncology nurses, and one laboratory technologist with the supervision of a senior nephrologist. Before the initiation of the data collection, the research team was trained on the objective of the study, the data collection tool, and how to use the electronic data system (Open Data Kit, ODK Collect) for two days. The collected data was checked for its consistency and completeness by the supervisor during the entire data collection period and necessary interventions were made to maintain the quality of data. The electronic data was then exported into SPSS software version 25.0 for data management and analysis. Before beginning the data analysis step, data was managed through data cleaning, transformation, and imputations.

### Statistical analysis

Participants’ sociodemographic and clinical characteristics were presented using frequencies and proportions. The GFR estimated by the different equations was summarized using mean ± standard deviation (SD) if it had a normal distribution, or median with 25th and 75th interquartile range (IQR) if the distribution was skewed. Normality was assessed using the Kolmogorov-Smirnov and Shapiro-Wilk tests, where a p-value > 0.05 indicated a normal distribution. To compare the underlying characteristics of the cases for which Cystatin C was measured and those for which it was not, we performed a chi-square test (or Fisher’s exact test where the assumptions of the chi-square test were not met) at 5% level of significance.

In order to evaluate the level of agreement between the 12 GFR estimation equations and the Cockcroft-Gault method, bias, precision, and accuracy were analyzed for each equation. Bias, or mean error (ME), was estimated by calculating the difference in eGFR value between the Cockcroft-Gault and the estimation equations and presented with its upper and lower ranges using 95% limits of agreement. A result of 0 indicates perfect agreement, a negative value suggests overestimation by the equations, and a positive value indicates underestimation. Values closer to 0 (positive or negative) reflect lower bias, while those further from 0 reflect higher bias. Precision was measured with the standard deviation of the ME. Finally, accuracy was assessed using root-mean squared error (RMSE), which represents the square root of the mean of the errors (biases) that are squared. For both precision and accuracy, a value close to 0 indicates high precision and accuracy and far from 0 indicates low precision and accuracy. Therefore, an estimation equation can be considered to potentially have good agreement with Cockcroft-Gault if it exhibits minimal bias, high precision, and high accuracy.

While minimal bias, high precision, and high accuracy suggest an estimation equation might have good agreement with Cockcroft-Gault, further statistical analysis was carried out to confirm the results. First, a one-sample t-test, at 5% significance level, was performed for each equation to assess if the observed bias is statistically significant. A p-value ≤ 0.05 indicates a significant difference, ruling out agreement. Conversely, a p-value > 0.05 suggests a non-significant difference and hence a potential agreement, requiring further evaluation with a Bland-Altman plot. The plot visualizes the distribution of the mean of the Cockcroft-Gault GFR and eGFR plotted against the difference between the two. The presence of some pattern in the distribution (heteroscedasticity) suggests the presence of proportional bias, indicating no agreement. On the other hand, a random scatter pattern signifies consistent differences across measurements, potentially indicating good agreement (no proportional bias). Finally, for equations with a suggestive Bland-Altman plot, a linear regression analysis was run at 5% level of significance, where a p-value ≤ 0.05 indicates significant difference and hence a lack of agreement, while a non-significant result (p-value > 0.05) confirms the presence of good agreement.

### Ethical considerations

The study was conducted after securing ethics approval from the institutional review board of St. Paul’s Hospital Millennium Medical College (SPHMMC-IRB) (Ref. No. PM23/367). Written informed consent was obtained from the participants. Participants’ confidentiality and privacy was maintained throughout the study period. Anonymity of the participants was maintained by use of medical record number in the research report. No other personal identifiers of the patients were used in the research report. Access to the collected information was limited to the investigators and confidentiality was maintained throughout the project.

## Result

### Socio-demographic and clinical characteristics

More than two-thirds of the participants were older than 40 years old, with over half (42.6%) falling between the ages of 40 and 59 and a quarter (25.0%) being 60 or older. Additionally, 94 (69.1%) were female. Of the participants, 22 (16.2%) fell into the underweight category, 33 (24.3%) were overweight, and only four (2.9%) were classified as obese.

Nearly a quarter (24.3%) had at least one underlying chronic medical illness. Hypertension was the most prevalent, affecting 11 participants (8.1%), followed by type 2 diabetes mellitus documented in 6 participants (4.4%). Breast cancer was the most common primary malignancy diagnosed in 63 (46.3%) participants, followed by colorectal cancer diagnosed in 22 (16.2%) participants.

The vast majority of participants received alkylating chemotherapeutic agents (91.2%), with immunotherapy administered in only three cases (2.2%). The majority of the participants (97.1%) had exposure to nephrotoxic agents (Table 1).

**Table 1:** Sociodemographic and clinical characteristics of cancer patients undergoing assessment for first-line chemotherapy at a tertiary hospital oncology center in Ethiopia, November 1, 2021 to April 30, 2022 (n = 136).

Variables	Category	Frequency	Percentage
**Age group (in years)**	<40	44	32.4
	40-59	58	42.6
	≥ 60	34	25.0
**Sex**	Male	42	30.9
	Female	94	69.1
**BMI**	Underweight	22	16.2
	Normal	77	56.6
	Overweight	33	24.3
	Obese	4	2.9
**Chronic medical illness**	No	103	75.7
	Yes	33	24.3
	Hypertension	11	8.1
	Type 2 Diabetes Mellitus	6	4.4
	HIV	4	2.9
	Anemia	4	2.9
	CKD	3	2.2
	Asthma	3	2.2
	Pulmonary tuberculosis	1	0.7
	DVT/PTE	1	0.7
**Primary malignancy**	Breast cancer	63	46.3
	Colorectal cancer	22	16.2
	Pancreatic cancer	8	5.9
	Head & neck cancer	8	5.9
	Bone and soft tissue cancer	6	4.4
	Stomach cancer	5	3.7
	Lung cancer	4	2.9
	Prostatic cancer	4	2.9
	Bladder cancer	3	2.2
	Cholangiocarcinoma	3	2.2
	Esophageal cancer	2	1.5
	Skin cancer	1	0.7
	Lymphoma	1	0.7
	Thyroid cancer	1	0.7
	Endometrial cancer	1	0.7
	Anal and perianal cancer	1	0.7
	Periampullary cancer	1	0.7
	Penile Cancer	1	0.7
	Thymoma	1	0.7
**Chemotherapy**	Alkylating agent	124	91.2
	Antitumor antibody	72	52.9
	Antimetabolites	51	37.5
	Plant alkaloid	38	27.9
	Topoisomerase inhibitors	2	1.5
**Immunotherapy**	No	133	97.8
	Yes	3	2.2
**Nephrotoxic drug exposure**	No	4	2.9
	Yes	132	97.1

**N.B:** BMI = Body mass index, HIV = Human Immunodeficiency virus, CKD = Chronic kidney disease, DVT = Deep venous thrombosis, PTE = Pulmonary thromboembolism.

### Agreement between GFR estimation equations and GFR estimated by Cockcroft-Gault

The results of the 12 eGFR equations showed that MDRD, MDRDef, CKD-EPI 2009 Cref, CKD-EPI 2021, FAS Cr, and FAS Craf overestimated the measured GFR. Conversely, CKD-EPI 2009 Cr, CKD-EPI 2012 CysC, CKD-EPI 2021 Cr-CysC, FAS CysC, FAS Cr-CysC, and FAS Cr-CysCaf underestimated the GFR. However, a comparison of these estimations’ performance with Cockcroft-Gault using a one-sample t-test revealed no significant difference in eGFR for MDRD, CKD-EPI 2009 Cr, CKD-EPI 2021, FAS Cr, and FAS Cr-CysCaf. Accordingly, the formula with the least bias was CKD-EPI 2009 Cr (ME = 0.72 ml/min, 95% CI: −67.66, 69.10 ml/min), followed by CKD-EPI 2021 (ME = −1.39 ml/min, 95% CI: −76.52, 73.74 ml/min), MDRD (ME = −4.07 ml/min, 95% CI: −82.70, 74.56 ml/min), FAS Cr (ME = −6.37 ml/min, 95% CI: −83.49, 70.75 ml/min), and FAS Cr-CysCaf (ME = 7.52 ml/min, 95% CI: −58.96, 73.99 ml/min). Furthermore, the precision and accuracy analysis showed that FAS Cr-CysCaf demonstrated the highest precision (SD = 33.92) and accuracy (RMSE = 34.53), followed by CKD-EPI 2009 Cr (SD = 34.89, RMSE = 34.89), CKD-EPI 2021 (SD = 38.33, RMSE = 38.21), FAS Cr (SD = 39.35, RMSE = 39.35), and MDRD (SD = 40.12, RMSE = 40.17) (Table 2).

**Table 2:** Agreement between GFR estimation equations and GFR estimated by Cockcroft-Gault among cancer patients undergoing assessment for first-line chemotherapy at a tertiary hospital oncology center in Ethiopia, November 1, 2021 to April 30, 2022 (n = 136).

Estimated GFR models	GFR (in ml/min/1.73 m2)	Bias		Precision (SD of bias)	Accuracy (RMSE)	p-value
		ME	95% limits of agreement			
**Cockcroft-Gault** (n = 136)	104.30 (78.9, 121.35)					
**MDRD** (n = 136)	107.85 (90.13, 133.55)	−4.07	−82.70, 74.56	40.12	40.17	0.239
**MDRDef** (n = 136)	130.70 (109.15, 161.88)	−27.54	−111.61, 56.54	42.90	50.84	<0.0001*
**CKD-EPI 2009 Cr** (n = 136)	110.00 (97.73, 118.15)	0.72	−67.66, 69.10	34.89	34.77	0.809
**CKD-EPI 2009 Cref** (n = 136)	127.5 (113.23, 136.95)	−16.19	−84.44, 52.07	34.82	38.29	<0.0001*
**CKD-EPI 2012 CysC** (n = 81)	81.26 ± 19.37	25.11	−48.09, 98.31	37.35	44.81	<0.0001*
**CKD-EPI 2021 Cr-CysC** (n = 81)	95.56 ± 16.99	10.81	−57.99, 79.61	35.10	36.52	0.007*
**CKD-EPI 2021**	111.85 (101.15, 118.58)	−1.39	−76.52, 73.74	38.33	38.21	0.672
**FAS Cr** (n = 136)	113.57 ± 29.14	−6.37	−83.49, 70.75	39.35	39.72	0.061
**FAS Craf** (n = 136)	126.95 ± 31.08	−19.75	−93.06, 53.56	37.40	42.18	<0.0001*
**FAS CysC** (n = 81)	83.02 ± 18.67	23.35	−47.80, 94.50	36.30	42.97	<0.0001*
**FAS Cr-CysC** (n = 81)	94.57 ± 18.48	11.80	−54.13, 77.74	33.64	35.45	0.002*
**FAS Cr-CysCaf** (n = 81)	98.85 ± 19.45	7.52	−58.96, 73.99	33.92	34.53	0.051

**N.B:** * = statistically significant.

**Fig 1:**
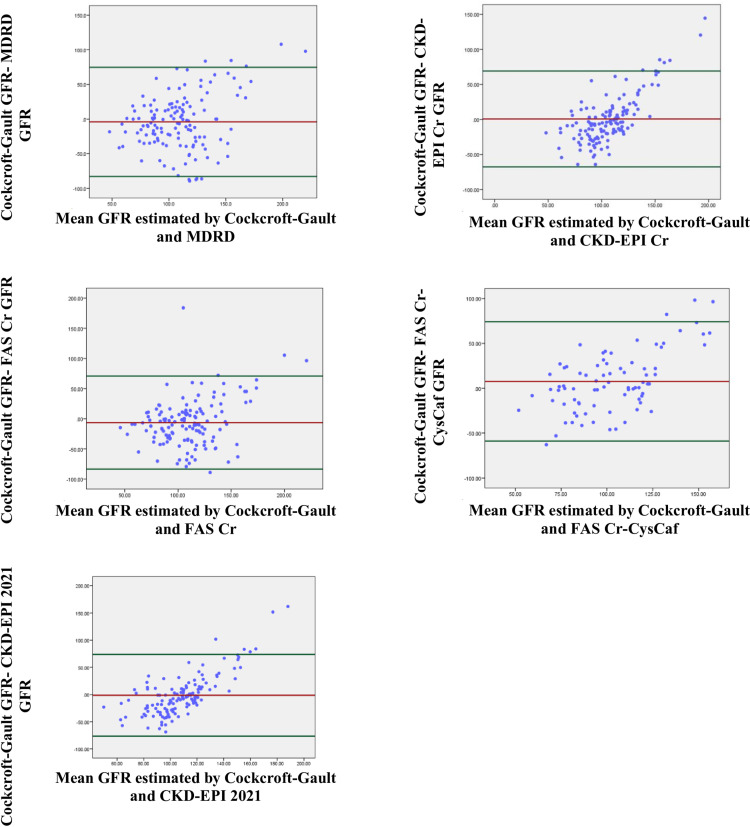
Bland-Altman Plots of GFR estimation equations and GFR estimated by Cockcroft-Gault among cancer patients undergoing assessment for first-line chemotherapy at a tertiary hospital oncology center in Ethiopia, November 1, 2021 to April 30, 2022.

To further assess the agreement between the five measurements and Cockcroft-Gault, a Bland-Altman plot was generated. The plots revealed a heteroscedastic result with varying variance, indicating the presence of proportional bias for all the measurements (Fig 1). This finding was further supported by the linear regression analysis, where all measurements showed statistically significant differences from the Cockcroft-Gault estimation: MDRD (β = 0.436, p < 0.0001), CKD-EPI 2009 Cr (β = 1.057, p < 0.0001), CKD-EPI 2021 (β = 1.264, p < 0.0001), FAS Cr (β = 0.432, p < 0.0001), and FAS Cr-CysCaf (β = 0.843, p < 0.0001) indicating the lack of agreement (Table 3).

**Table 3:** Linear regression results to assess agreement between GFR estimation equations and GFR estimated by Cockcroft-Gault among cancer patients undergoing assessment for first-line chemotherapy at a tertiary hospital oncology center in Ethiopia, November 1, 2021 to April 30, 2022 (n = 136).

Estimated GFR models	Crude regression coefficient (β)	95% Confidence Interval for β	p-value
**Cockcroft-Gault**			
**MDRD**	0.436	0.209, 0.662	<0.0001*
**CKD-EPI 2009 Cr**	1.057	0.898, 1.215	<0.0001*
**CKD-EPI 2021**	1.264	1.079, 1.448	<0.0001*
**FAS Cr**	0.432	0.212, 0.652	<0.0001*
**FAS Cr-CysCaf**	0.843	0.597, 1.089	<0.0001*

**N.B:** β = Regression coefficient, * = statistically significant.

## Discussion

The study aimed to assess the agreement between creatinine- and cystatin-C-based GFR estimation equations and GFR estimated by Cockcroft-Gault for appropriate chemotherapy drug dosing among 136 cancer patients screened for initiation of first-line chemotherapy at a tertiary hospital oncology center in Ethiopia. The majority of the patients were older than 40 years (67.6%) and female (69.1%). Close to half (43.4%) of the patients had abnormal BMI, and 24.3% had pre-existing conditions, mainly hypertension (8.1%) and type 2 diabetes mellitus (4.4%). Breast cancer (46.3%) and colorectal cancer (16.2%) were the most common primary malignancies diagnosed, and alkylating agents were the primary chemotherapeutic drugs used (97.1%).

The comparison of the 12 eGFR equations with Cockcroft-Gault revealed variation, with some methods underestimating and others overestimating GFR. However, only five equations showed potential agreement with Cockcroft-Gault based on a one-sample t-test: MDRD, CKD-EPI 2009 Cr, CKD-EPI 2021, FAS Cr, and FAS Cr-CysCaf. Among these, CKD-EPI 2009 Cr exhibited the least bias (ME = 0.72 ml/min, 95% CI: −67.66, 69.10 ml/min), while FAS Cr-CysCaf demonstrated the highest precision (SD = 33.92) and accuracy (RMSE = 34.53). However, further analysis using Bland-Altman plots and linear regression to confirm agreement revealed no agreement between any of the formulas and Cockcroft-Gault. This shows that the more recent equations, generally considered more accurate, including the currently recommended formula for cancer patients, CKD-EPI 2021, lacked agreement with Cockcroft-Gault [4,8,15]. This is concerning because Cockcroft-Gault has limitations, particularly in cancer patients, who often face a number of factors that can compromise GFR accuracy, including older age, dehydration, reduced muscle mass, and underlying chronic illnesses like chronic kidney disease, all of which can further impair kidney function. Narrow therapeutic indices of the chemotherapeutic drugs also require a more accurate GFR estimation equation for the better treatment outcomes. Studies that assessed the performance of different GFR estimation equations, including Cockcroft-Gault, as compared to the standard GFR estimation also revealed that CKD-EPI 2009 and MDRD are better than Cockcroft-Gault, with CKD-EPI 2009 showing superior performance in cancer patients [27,28]. This lack of agreement suggests that the current practice of GFR estimation using Cockcroft-Gault formula may not be ideal for cancer patients, potentially leading to under- or overestimation of chemotherapy drug dosing and ultimately ineffective treatment, complications, and worse patient outcomes.

This study provides valuable insights into the accuracy of GFR estimation methods among cancer patients in the study setting. However, the following limitations restrict the generalizability of the findings. A significant limitation arises from the overall small sample size of the study and the further reduced sample size for GFR estimation equations utilizing Cystatin C, potentially limiting the statistical power and introducing bias. Furthermore, the absence of a gold standard GFR measurement within the study population precludes direct comparison of estimated GFR values with actual GFR, limiting the ability to definitively assess the accuracy and precision of the different estimation equations. Finally, the study focuses solely on cancer patients, limiting the ability to compare the findings with a healthy population. These limitations should be carefully considered when interpreting the study findings and drawing conclusions.

## Conclusion

The study revealed that the most recent and accurate GFR estimation equations that are recommended to be used in cancer patients did not show agreement with Cockcroft-Gault, the formula that is widely used for practice in Ethiopia. This suggests that current GFR estimation practices in cancer patients might be inaccurate, potentially leading to improper chemotherapy dosing and poorer patient outcomes. This emphasizes the need for further large, multicenter studies to identify the most accurate eGFR equation for this specific patient population in comparison with actual GFR measurement for safer and more effective chemotherapy dosing strategies.

## Supporting information

S1 Deidentified data. (CSV)
